# *Salmonella* Derby adaptation to swine and simultaneous attenuation for humans go through decay of *Salmonella* Pathogenicity Island I

**DOI:** 10.1128/spectrum.01899-23

**Published:** 2023-10-06

**Authors:** Melissa Berni, Luca Bolzoni, Ilaria Menozzi, Alessandra Dodi, Chiara Bracchi, Marina Morganti, Erika Scaltriti, Stefano Pongolini, Martina Tambassi

**Affiliations:** 1 Risk Analysis and Genomic Epidemiology Unit, Istituto Zooprofilattico Sperimentale della Lombardia e dell'Emilia-Romagna (IZSLER), Parma, Italy; Universidad Andres Bello, Santiago, Chile

**Keywords:** *Salmonella*, host-adaptation, *Salmonella* pathogenicity island I

## Abstract

We report the identification, within the global population of *Salmonella* Derby, of a lineage highly diffused and expanding in swine while being significantly under-represented in humans which carries stop mutations in the Salmonella Pathogenicity Island 1 (SPI-1) genes *sipA* and *hilC*. Single-cell analysis of invasion, vacuolar load, and cytosolic hyper-replication show that these mutations attenuate *S*. Derby virulence in human intestinal cells. Identification of this lineage follows the previous detection of another lineage over-represented in swine compared with humans which carries a loss-of-function mutation in *hilD*, the major SPI-1 regulator, responsible for reduced infection of human cells. Notably, this *hilD*-mutated lineage represents a sub-population of the wider *sipA* and *hilC-*mutated lineages identified in this study, indicating that SPI-1 is subjected to genetic decay in this part of the *Salmonella* Derby population adapted to swine. Virulence assessment on swine intestinal cells showed drastically lower levels for both wild-type and mutant *S*. Derby compared with human cells. Still, *S*. Derby with impaired *sipA* and *hilC* showed further attenuation also in swine cells compared with the wild type. These data suggest that invasion of swine intestinal epithelium is not needed by *S*. Derby to circulate in pigs, leading to the decay of SPI-1, which in turn determines attenuation for humans. Accordingly, a further *S*. Derby lineage under-represented in humans compared with pigs was found carrying truncated or missing SPI-1 genes, indicating that the *S*. Derby population is undergoing converging evolution in its adaptation to swine while attenuating for humans through loss of SPI-1 function.

**IMPORTANCE:**

This study integrated population data with *in vitro* assessment of virulence phenotypes to unveil that a considerable part of the global population of *Salmonella* Derby is evolving to enhance its host adaptation to the swine host and that this evolution is simultaneously increasing its attenuation for humans. The study shows that the fixation of deleterious mutations in SPI-1 has a role in this process. This evidence indicates that SPI-1 has a key role for *S*. Derby virulence in humans but not for its circulation in swine. The results show that genes generally considered essential for *Salmonella* pathogenesis do not play the same key role for all *Salmonella* serovars or lineages and/or all hosts. The study helps in understanding the molecular mechanisms underlying the ecology and host adaptation of *Salmonella* showing that the adaptation process can vary for different types of *Salmonella* and hosts.

## INTRODUCTION

*Salmonella* is a major foodborne pathogen worldwide, the second most reported agent of zoonotic diseases, and the first cause of foodborne outbreaks in Europe (1). The main source of salmonellosis is food of animal origin as *Salmonella* infects many animal species as well as humans. In particular, pork is a major vehicle of salmonellosis, because of its considerable consumption and the high prevalence of swine infection (2). The mostly isolated *Salmonella enterica* serovars from pigs in the EU are *S*. Typhimurium (15.3%), monophasic variant of *S*. Typhimurium (28.2%), and Derby (22.3%). Although similarly present in pigs, these serovars differ substantially in their zoonotic potential. *S*. Typhimurium and monophasic *S*. Typhimurium cause 11.4% and 8.8% of salmonellosis cases in humans, respectively, whereas *S*. Derby is responsible for only 0.93% of human cases (1). This is consistent with the established knowledge that *S*. Typhimurium and its monophasic variant are generalist serovars infecting multiple hosts, while *S*. Derby is a swine-adapted serovar commonly found in pigs but only rarely detected in humans (3, 4). This unbalance in the incidence of infection by *S*. Derby in swine compared with humans offers the opportunity to investigate the molecular mechanisms of *Salmonella* adaptation. In a previous study, we identified a specialized case of host adaptation to swine by a specific *S*. Derby lineage, featuring even further decreased pathogenicity to humans while maintaining high prevalence in pigs (5). We found that the lineage carries a single loss-of-function mutation in *hilD*, encoding the master activator of *Salmonella* Pathogenicity Island 1 (SPI-1) (6), and demonstrated that the *hilD* mutation was responsible for impaired interaction with human cells much more than with swine cells, potentially explaining the negligible presence of this lineage in humans despite its circulation in swine. This finding represents an example of *Salmonella* evolution towards host adaptation through the generation of allelic variants by point mutation, a reported mechanism of host adaptation along with genome degradation and horizontal gene transfer (7). SPI-1 is a ~40-kb genomic region which includes genes encoding a Type 3 Secretion System (T3SS), effector proteins, and transcriptional regulators of SPI-1 genes (8). The SPI-1 products are considered essential for each step of *Salmonella* pathogenesis inside intestinal epithelial cells, i.e., invasion, survival in *Salmonella*-containing vacuole (SCV), replication inside SCV and hyper-replication into the cytosol of cells (9).

The relatively low impact of the observed *hilD* mutation on the interaction of *S*. Derby with swine cells compared with the high impact on human cells suggests that the mutation of SPI-1 genes could be a molecular mechanism for *S*. Derby to further specialize for the swine host while losing pathogenicity for humans.

In line with this hypothesis, we further observed that the specific lineage with the loss-of-function mutation in *hilD* also carried stop mutations in the coding sequences of both *sipA*, encoding the SPI-1 virulence effector SipA, and *hilC*, encoding the SPI-1 minor transcriptional activator HilC.

SipA is a multifunctional protein that takes part in each step of the enterocyte colonization, i.e., enterocyte invasion, vacuolar survival and replication, and cytosolic hyper-replication (10 – 12). Once inside epithelial cells, SipA is cleaved by cellular caspase-3 into two functional domains: the actin-binding C-terminal domain (SipA^426-685^) involved in cell invasion (13) and the N-terminal domain (SipA^1-425^) involved in *Salmonella* survival inside the SCV and in gut mucosa inflammation, by stimulating trans-epithelial migration of polymorphonuclear neutrophils (14 – 16). The observed stop codon in *sipA* is located at the amino acid position 350, resulting in a truncated N-terminal domain.

HilC is an AraC/XylS transcriptional regulator that, together with the other AraC/XylS regulators HilD and RtsA, controls expression of the master SPI-1 activator *hilA* (6, 17). The system is controlled primarily by HilD, whereas HilC and RtsA amplify and accelerate SPI-1 gene expression (18). HilC has a two-domain structure comprising an N-terminal regulatory domain and a C-terminal highly conserved DNA-binding domain with two helix-turn-helix motifs corresponding to amino acid intervals 212–233 and 259–282 (UniProtKB entry E1WAB2). The observed stop codon in *hilC* is located at amino acid position 209 of 295 in the region encoding the C-terminal DNA-binding domain, upstream of the first helix-turn-helix motif.

To contribute to the understanding of the molecular mechanisms of *S*. Derby host adaptation, we investigated the diffusion of the observed mutations in SPI-1 genes in the global population of available genomes of *S*. Derby from swine (ca. 1490 genomes) as reported in the Enterobase database. Furthermore, we assessed the impact of such mutations on virulence by quantifying invasion, vacuolar load, and cytosolic hyper-replication in both human and swine intestinal epithelial cells, as these *in vitro* phenotypes have been associated with different pathological outcomes *in vivo*. In particular, *Salmonella* survival inside the SCV has been associated with systemic and enduring infections (19, 20) while the cytosolic hyper-replication triggers *Salmonella* intestinal expansion and gut inflammation (21, 22).

Moreover, we searched for other mutations in the SPI-1 genes of available *S*. Derby genomes and evaluated if multiple *S*. Derby lineages underwent converging evolution in their adaptation to swine through SPI-1 loss of function.

## RESULTS

### *S*. Derby-carrying stop mutations in *sipA* and *hilC* represent a diffused and expanding lineage in pigs while remaining rare among humans

**Fig 1:**
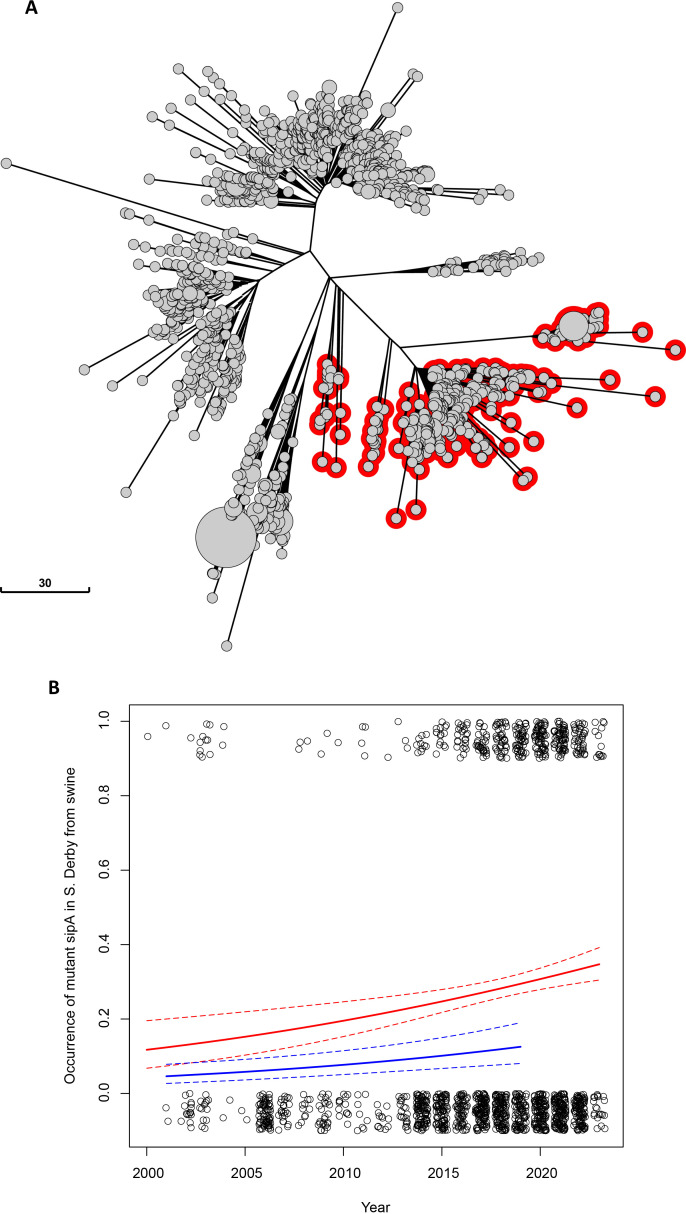
The *hilC*_stop_ and *sipA*_stop_-carrying lineage in the context of the *S*. Derby population from swine and its occurrence over time in this host. Analysis was performed on 1,490 genomes of *S*. Derby ST40 isolated in swine in Europe and North America in the 2000–2023 time period available in Enterobase (accessed date 7 April 2023). (**A**) Neighbor-joining tree based on *S. enterica* cgMLST allelic profiles. Nodes encircled in red represent the *S*. Derby genomes carrying the stop mutations in *sipA* and *hilC* genes. (**B**) Occurrence of *S*. Derby genomes displaying the stop mutations in *sipA* and *hilC* genes (open dots) as a function of time, i.e., isolation year. The solid red and blue lines represent the fraction of genomes over time displaying the stop mutations in North America and Europe, respectively, estimated through the logistic regression model. Dashed lines represent the confidence intervals of the estimates.

To evaluate the abundance of the described stop mutations in *sipA* and *hilC* in the global population of *S*. Derby, we analyzed a set of 1,490 *S*. Derby genomes of swine origin from Europe and North America (2000–2023 time period) belonging to Sequence Type 40 (ST40) available in the Enterobase database. Sequence type ST40 represents the most diffused ST of *S*. Derby in swine (4). We found that 24.9% of the ST40 genomes from swine carried both the stop mutations in *sipA* (*sipA*_stop_ allele) and *hilC* (*hilC*_stop_ allele). No isolate was found with only one of the two mutations. Moreover, the neighbor-joining tree of the ST40 genomes from swine, generated under the Enterobase cgMLST v2 HierCC v1 scheme (23), highlighted that all the genomes carrying the *sipA* and *hilC* mutations represented different branches of a single large lineage of the population (see red-encircled nodes in Fig. 1A). This *hilC*_stop_ and *sipA*_stop_-carrying lineage included the 15 isolates carrying also the loss-of-function mutation in *hilD* previously characterized (5). The logistic regression performed on the occurrence of the genomes carrying *sipA*_stop_ and *hilC*_stop_ in the ST40 population from swine as a function of time (considering also the continent of isolation) showed a significant increase of the fraction of mutation-carrying genomes over time, both in Europe and North America (Fig. 1B). Moreover, the logistic regression showed that the proportion of mutated genomes was significantly higher in North America (28.4%) compared with Europe (8.4%), *P* < 0.0001. Furthermore, using the data from Enterobase, we compared the proportion of ST40 genomes carrying the mutations isolated from swine with that observed in the 706 ST40 genomes isolated from humans in Europe and North America in the same period. We found that the proportion in humans was significantly lower than that in pigs (*P* < 0.0001) both in Europe (4.3% vs 8.4%) and North America (12% vs 28.4%).

### The stop mutation in *hilC* does not alter SPI-1 expression

The stop mutation in *hilC* generated an activator unable to do its function because of the loss of its DNA-binding domain; therefore, the mutation corresponded to the loss of the entire *hilC* gene. To assess the effect of the loss of *hilC*, we analyzed the expression of SPI-1 genes in strain ER1175, naturally carrying the wild-type *S*. Derby alleles of *hilC* and *sipA*, ER1175 deleted for *hilC* (ER1175Δ*hilC*) and the strain N11, a representative of the group of isolates naturally carrying *hilC*_stop_ and *sipA*_stop_. For the expression analysis, strains were grown to the early stationary phase as it was previously reported that in this phase, SPI-1 is highly transcribed and significantly downregulated in ER1175 deleted for *hilD* (encoding the SPI-1 master activator) compared with the wild-type strain (5, 24). The expression of SPI-1 genes was not significantly reduced in ER1175Δ*hilC* and N11 compared with ER1175, indicating that the loss of *hilC* does not have an appreciable impact on SPI-1 expression in the tested conditions (Table S1). These data are in line with the already reported minor role of HilC in the activation of SPI-1 expression (18).

### The stop mutations in *sipA* and *hilC* impair the ability of *S*. Derby to infect human cells

**Fig 2:**
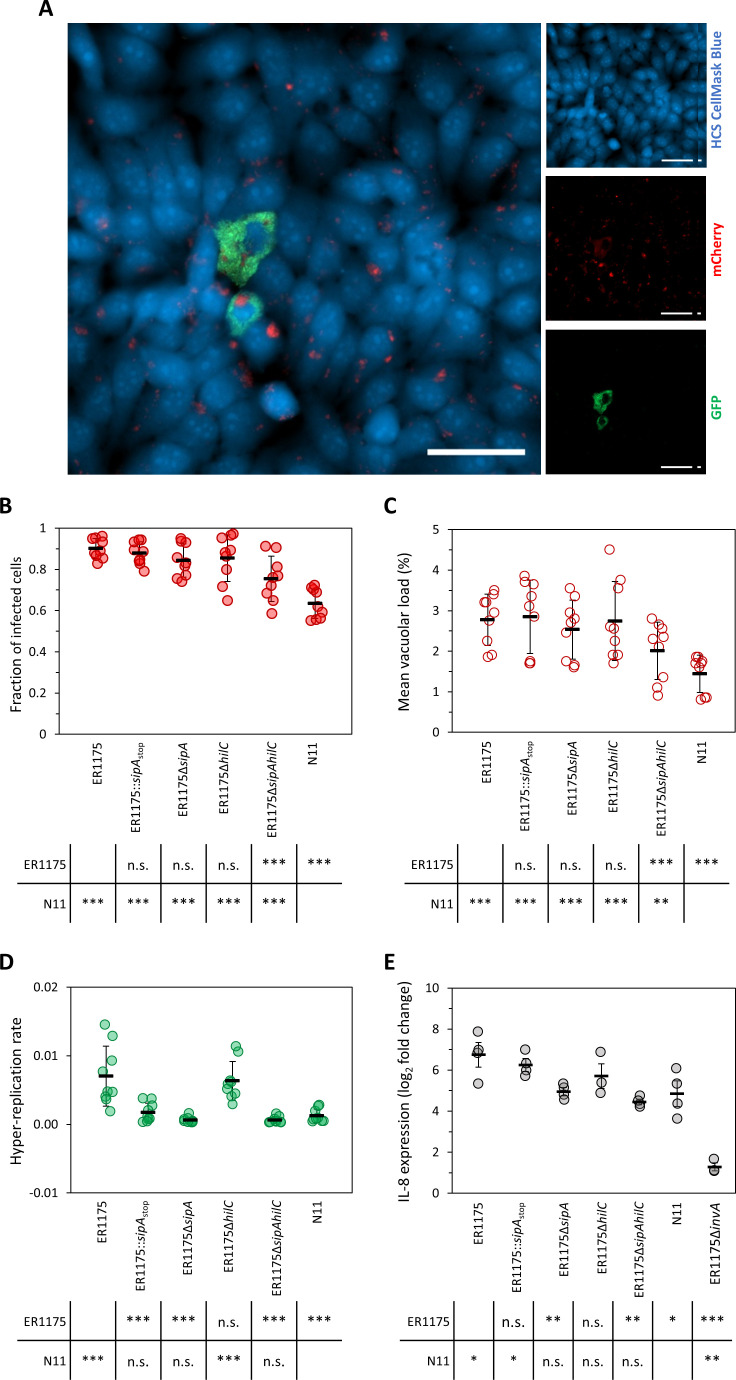
Derby interaction with human epithelial cells. (**A**) Representative image of INT-407 cells infected with *S*. Derby ER1175 carrying the pCHAR-Duo fluorescence reporter plasmid. Epithelial cells are shown in blue (HCS CellMask Blue), intracellular *Salmonella* in red (mCherry), and cytosolic hyper-replicating *Salmonella* in green (GFP). White scale bars are 50 µm. The extent of invasion, vacuolar load and cytosolic hyper-replication for ER1175, its isogenic mutants, and N11 was calculated. (**B**) The fraction of infected cells was obtained by dividing the number of cells with a percentage of area occupied by mCherry-only expressing *Salmonella* > 0.2% by the total number of cells. (**C**) The mean vacuolar load was obtained by calculating the mean percent area occupied by mCherry-only expressing *Salmonella* in the infected cells. (**D**) The hyper-replication rate was calculated by dividing the number of cells with a percentage of the area occupied by GFP-expressing *Salmonella* ≥ 20% by the total number of infected cells. Data shown in B, C, and D are the pooled data of three biological replicates with three technical replicates. Horizontal bars indicate the mean of biological replicates. Vertical bars indicate standard deviation. Asterisks indicate significant difference versus ER1175. (**E**) IL-8 expression of human cells infected with *S*. Derby strains analyzed. Each dot represents the log2 fold change of IL-8 expression of infected versus non-infected cells for each biological replicate. Data from three to four biological replicates are reported. Horizontal bars indicate the mean of biological replicates. Vertical bars indicate the standard error of the mean. Tables report *P*-values from the *t*-statistics using Satterthwaite’s method with Bonferroni post-hoc correction (*P < 0.05, **P < 0.01, and ***P < 0.001; n.s., not significant).

Based on the limited diffusion of *hilC*_stop_ and *sipA*_stop_-carrying *S*. Derby among humans despite its considerable and increasing circulation in swine, we hypothesized that the two stop mutations in *sipA* and *hilC* could be associated with possible weakening of the infection mechanisms in the human host and thereby explain the reduced risk for this species observed at population scale. We thus performed cell culture infection assays on human-derived INT-407 intestinal epithelial cells. Automated fluorescence microscopy was used to quantify at the single cell level the extent of invasion, vacuolar load, and cytosolic hyper-replication (25) (Fig. 2A). Strain ER1175, naturally carrying the wild-type *S*. Derby alleles of *hilC* and *sipA*, and strain N11, a representative of the *hilC*_stop_ and *sipA*_stop_-carrying *S*. Derby, were tested together with ER1175 mutants generated to analyze the impact of each mutation, singularly or in association, on the infection of human cells. The reference strain *S*. Typhimurium SL1344 was included in the analysis as positive control of infection (Fig S1).

The extent of invasion was calculated as the fraction of infected cells, namely, the proportion of infected cells with ≥0.2% of their area occupied by *Salmonella*. N11 invaded a significantly lower fraction of cells than ER1175 (0.63 vs 0.90) (Fig. 2B). To determine the specific effect of the stop mutation in *sipA* on invasion, an ER1175 mutant carrying *sipA*_stop_ was produced (ER1175::*sipA*_stop_). Also, the deletion mutant ER1175Δ*sipA* was included in the analysis to evaluate if the *sipA* stop mutation, causing the loss of expression of the C- terminal domain specifically involved in invasion, conferred the same phenotype as the loss of the entire gene. The results showed that the introduction of *sipA*_stop_, as well as the deletion of *sipA*, did not significantly reduce the fraction of cells infected by ER1175. These results are consistent with the SipA cooperative and redundant role in promoting *Salmonella* entrance into the host cell (26). The effect of the stop mutation on *hilC*, corresponding to the loss of the entire gene, was assessed testing the invasion ability of ER1175Δ*hilC*. The deletion of *hilC* did not significantly reduce invasion compared with that of wild-type ER1175, in line with the already observed minor effect of HilC on activating SPI-1 expression (Table S1) and with the mild effect of the mutation of *hilC* on *S*. Typhimurium invasion (27). A mutant deleted for both *sipA* and *hilC* (ER1175Δ*sipAhilC*) was then tested to evaluate the combined effect of losing both genes, and we observed that ER1175Δ*sipAhilC* infected a significantly lower fraction of cells compared with ER1175 (0.75 vs 0.90). This result, together with the reduced invasion of N11, indicates that only the double impairment of *sipA* and *hilC* can lead to reduced invasion ability, suggesting an additive effect of the two mutations on invasion.

The vacuolar load was quantified as the mean of the percentages of cellular areas occupied by vacuolar *Salmonella*. Two SPI-1-regulated events contribute to the magnitude of this phenotype, the internalization of multiple *Salmonella* in a SCV (28), and the survival and replication inside the SCV (29 – 31) (Fig. 2C). The same pattern observed for invasion was detected for the vacuolar load, with only ER1175Δ*sipAhilC* and N11 showing a significantly lower mean vacuolar load compared with ER1175 (2.01 and 1.44 vs 2.78). Moreover, strain N11 showed an invasion level and vacuolar load even lower than ER1175Δ*sipAhilC*, suggesting that also other genetic features of the *hilC*_stop_ and *sipA*_stop_-carrying *S*. Derby are involved in these phenotypes.

The cytosolic hyper-replication was scored as the proportion of infected cells massively colonized by cytosolic *Salmonella*, defined as infected cells with ≥20% of their area occupied by cytosolic *Salmonella*. Not only the double-mutation strains N11 and ER1175Δ*sipAhilC* but also the *sipA-*only impaired strains ER1175::*sipA*_stop_ and ER1175Δ*sipA* showed reduced ability to hyper-replicate in the cytosol of infected cells compared with ER1175 (Fig. 2D), in accordance with the known involvement of SipA in mediating intra-cytosolic survival and hyper-replication (12, 32). The same level of reduced hyper-replication observed for both ER1175::*sipA*_stop_ and ER1175Δ*sipA* demonstrates that the stop mutation in *sipA* generates an effector unable to exert its function. Conversely, ER1175Δ*hilC* showed no reduction of the hyper-replication rate compared with ER1175, consistently with the unaltered *sipA* expression observed in *hilC*-impaired strains ER1175Δ*hilC* and N11 (Table S1). In this case, N11 showed the same reduction in hyper-replication as *sipA-*only impaired strains, indicating that the stop mutation in *sipA* by itself is responsible for the lower ability of N11 to hyper-replicate in the cytosol of host cells.

Overall, these results showed that the two stop mutations of *sipA* and *hilC* reduce virulence of *S*. Derby in human cells and likely contribute to explain the reduced zoonotic risk observed in the population for the lineage carrying the two mutations.

### The truncated N-terminal domain of SipA retains the ability to induce IL-8 expression in human cells

The injection of SPI-1 effectors, and primarily SipA, inside intestinal epithelial cells is required for *Salmonella* to fully induce the expression of chemokines like IL-8 that stimulates the inflammatory response by inducing polymorphonuclear neutrophilic leukocyte migration, ultimately leading to enteritis and diarrhea (33, 34). We measured the level of IL-8 expression induced in human epithelial cells infected by ER1175, ER1175 mutants, and N11 to evaluate the impact of the stop mutations in s*ipA* and *hilC* on *S*. Derby ability to induce the inflammatory response (Fig. 2E). The *invA*-deleted mutant of ER1175 (ER1175Δ*invA*) was included in the analysis as negative control of IL-8 induction because *invA* encodes an essential component of the T3SS-1 (35) and its lack impairs the ability to inject SPI-1 effectors in the host cells and consequently to invade and induce expression of chemokines.

The infection with ER1175 induced the strongest increase in IL-8 expression (6.75-log_2_ fold change) in human cells as opposed to ER1175Δ*invA* which caused the weakest increase in IL-8 expression (1.28- log_2_ fold change), as expected. Compared with ER1175, ER1175::*sipA*_stop_ showed no significant defect in inducing IL-8 expression. This result indicates that the truncated N-terminal domain of SipA generated by the stop mutation retains the ability, albeit reduced, to induce the inflammatory response. Coherently, the effector encoded by *sipA*_stop_ still conserves part of the 131-amino acid region of the N-terminal domain (amino acids 294–424) involved in the induction of pro-inflammatory genes (14). The ER1175Δ*hilC* mutant showed no decrease in IL-8 induction compared with ER1175, which is consistent with the lack of any significant effects of *hilC* mutations on SPI-1 gene expression (Table S1). Otherwise, the expression of IL-8 in human cells infected by ER1175Δ*sipA* (4.95-log_2_ fold change) as well as ER1175Δ*sipAhilC* (4.45-log_2_ fold change) was significantly lower than that observed for ER1175, in line with the known role of SipA in inducing IL-8 expression. Overall, these results showed that the stop mutations in *sipA* and *hilC* individually have no significant effect on reducing the inflammatory response. N11 showed a significant defect in inducing IL-8 expression (4.85-log_2_ fold change) compared with ER1175. The reason for this behavior remains not fully explained, but it can be hypothesized that the copresence of the stop mutations in both *sipA* and *hilC* affects this phenotype. Notably, N11 was shown to have a lower invasion level (Fig. 2B) compared with ER1175 and a link between invasion defect and the diminished IL-8 induction has been reported (36).

### The enhanced adaptation to swine of *S*. Derby carrying *hilC*_stop_ *and sipA*_stop_ goes with the loss of virulence in swine enterocytes

**Fig 3:**
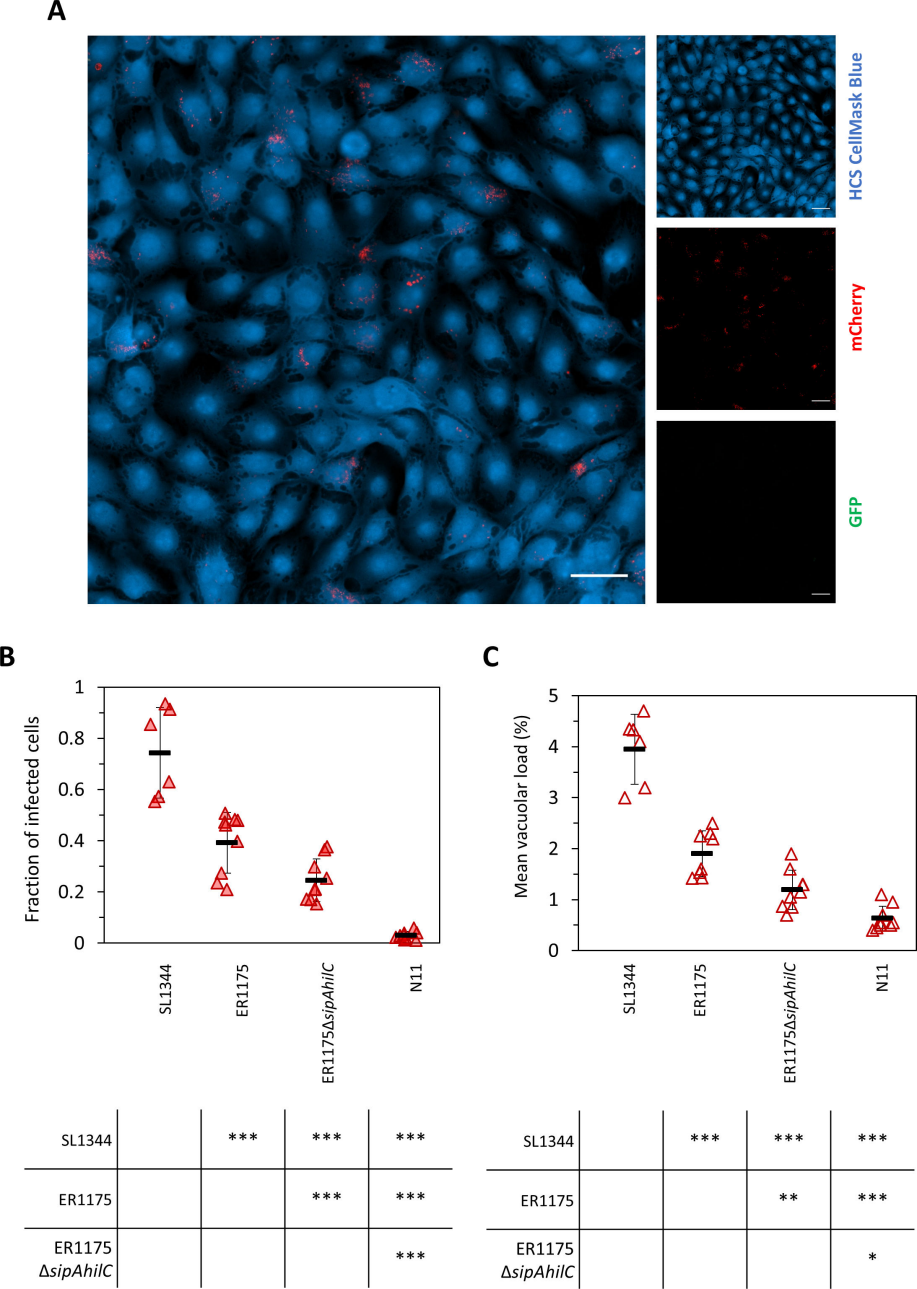
Derby interaction with swine epithelial cells. (**A**) Representative image of IPEC-J2 cells infected with *S*. Derby ER1175 carrying the pCHAR-Duo fluorescence reporter plasmid. Epithelial cells are shown in blue (HCS CellMask Blue), intracellular *Salmonella* in red (mCherry), and cytosolic hyper-replicating *Salmonella* in green (GFP). White scale bars are 50 µm. The extent of invasion and vacuolar load for *S*. Typhimurium SL1344 and *S*. Derby ER1175, ER1175Δ*sipAhilC*, and N11 was calculated. (**B**) The fraction of infected cells was obtained by dividing the number of cells with a percentage of the area occupied by mCherry-only expressing *Salmonella* > 0.2% by the total number of cells. (**C**) The mean vacuolar load was obtained by calculating the mean percent area occupied by mCherry-only expressing *Salmonella* in the infected cells. Data shown in B and C are the pooled data of two to three biological replicates with three technical replicates. Horizontal bars indicate the mean of biological replicates. Vertical bars indicate standard deviation. Tables report *P*-values from the *t*-statistics using Satterthwaite’s method with Bonferroni post-hoc correction (*P < 0.05, **P < 0.01, and ***P < 0.001).

*S*. Derby, a swine-adapted serovar, usually causes asymptomatic infection in swine, as opposed to the generalist serovar *S*. Typhimurium that can cause enterocolitic forms (37). This difference in pathogenesis *in vivo* between the two serovars is reflected in a reduced ability of *S*. Derby to invade and replicate in swine IPEC-J2 intestinal epithelial cells *in vitro* compared with *S*. Typhimurium (5). Therefore, we evaluated if *S*. Derby carrying *hilC*_stop_ and *sipA*_stop_, which appears well adapted to pigs considering its diffusion and expanding trend, was characterized by a further reduction in the ability to colonize swine enterocytes and if the reduction was due to the stop mutations in *sipA* and *hilC*. For this, the colonization steps of the swine IPEC-J2 intestinal epithelial cells were analyzed at a single-cell level for *S*. Typhimurium SL1344, ER1175, ER1175Δ*sipAhilC*, and N11 (Fig. 3A).

Comparing the different strains for their invasion ability in IPEC-J2 cells (Fig. 3B), a significant reduction was observed for all *S*. Derby strains compared with *S*. Typhimurium SL1344, with ER1175, ER1175Δ*sipAhilC*, and N11 infecting a fraction of swine cells of 0.39, 0.25, and 0.03, respectively, compared with 0.73 of *S*. Typhimurium. The lower invasion ability of ER1175Δ*sipAhilC* compared with ER1175 demonstrates that the two genes have a role in the infection of swine cells as well as in human cells. N11 infected swine cells even less than ER1175Δ*sipAhilC*, indicating that genetic signatures other than the stop mutations in *sipA* and *hilC* are involved in its phenotype.

The differences between the strains observed for invasion were reported also for the vacuolar load (Fig. 3C) with *S*. Typhimurium SL1344 presenting the highest vacuolar load, followed by ER1175, ER1175Δ*sipAhilC*, and N11 in decreasing order.

A strong reduction in the fraction of infected cells and in the vacuolar load was observed in swine cells compared with human cells for all *S*. Derby strains (−0.51 for ER1175, −0.50 for ER1175Δ*sipAhilC*, and −0.60 for N11). This can be partially explained by the fact that the IPEC-J2 cell line is more resistant to *Salmonella* infection, compared with human cell line (38). However, for *S*. Typhimurium SL1344, the difference in the fraction of swine vs human cells infected was only −0.24, indicating that the marked reduction observed for *S*. Derby strains was a serotype-specific feature. The mean vacuolar load, as well as the percentage of infected cells, was generally lower in swine cells compared with human cells.

The hyper-replication rate was not measurable for *S*. Derby strains in swine cells because of the low frequency of this phenotype along with the low infection rate in swine cells. We were able to quantify hyper-replication only for *S*. Typhimurium SL1344, which infected a percentage of swine cells much higher than S. Derby strains (Fig S2).

### Other lineages of *S*. Derby carry deleterious mutations in *sipA* and other SPI-1 genes

**Fig 4:**
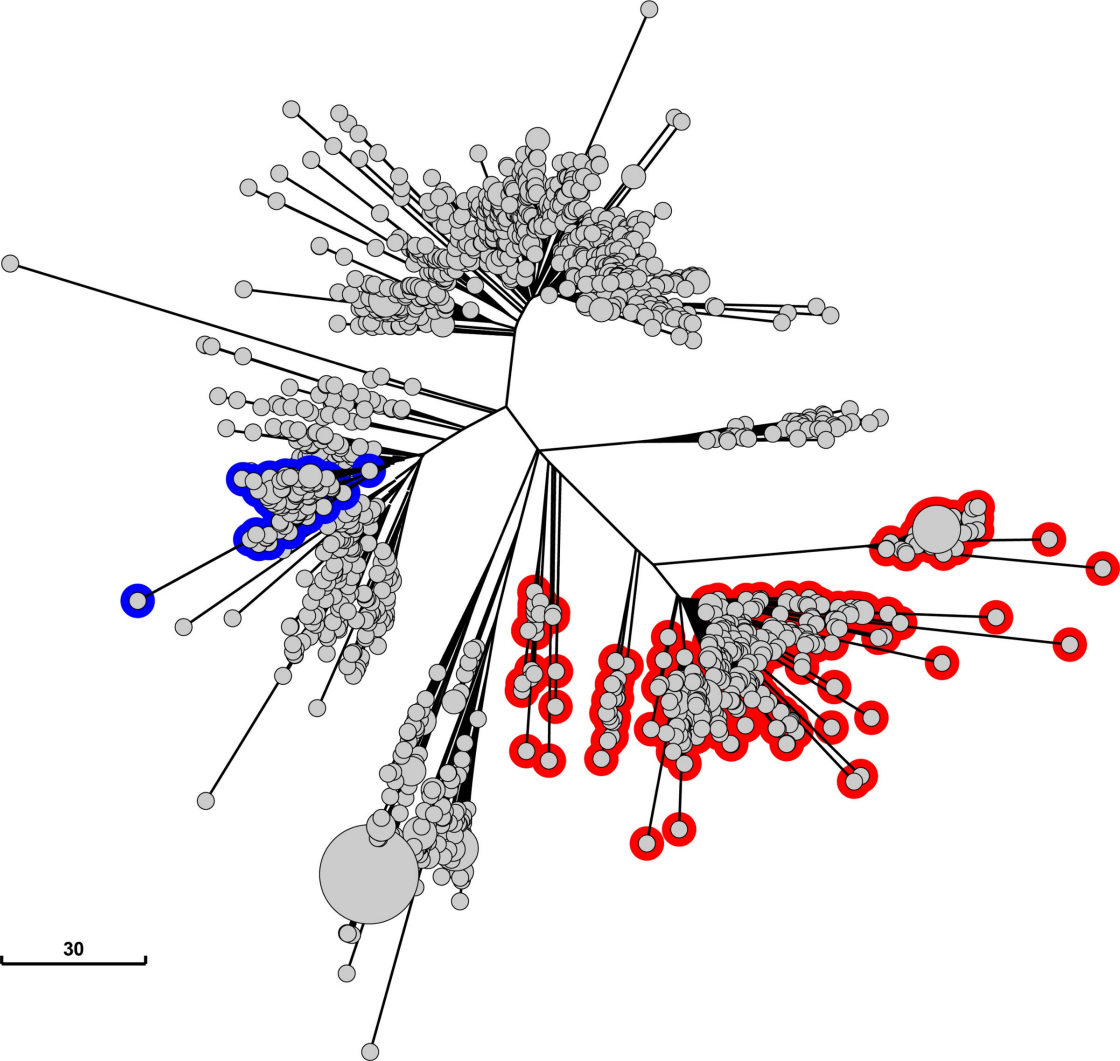
Neighbor-joining tree of the ST40 S. Derby genomes of swine origin employed under the cgMLST v2 HierCC v1 scheme. Circles represent *S*. Derby genomes analyzed. Genomes carrying the *sipA* and *hilC* stop mutations are highlighted in red; genomes with *sipA* truncated or missing are highlighted in blue.

Our results showed that the stop mutations in *sipA* and *hilC* reduced the virulence of *S*. Derby both in human and in swine cells. This attenuation is consistent with the limited proportion of human cases associated with this genotype; at the same time, it does not seem to negatively affect its circulation in swine. Under the hypothesis that this mutation process could be a more generalized phenomenon, we looked for other deleterious mutations in *sipA* and *hilC* in the framework of adaptation to swine. The same Enterobase data set of genomes used to identify the stop mutations in *sipA* and *hilC* was used for this search. No *hilC* alleles other than that described in this study were found carrying potentially deleterious mutations in the *S*. Derby ST40 population isolated from European and North American pigs. Conversely, 79 genomes (5.3% of the population) carrying a truncated (68 genomes) or missing (11 genomes) version of the *sipA* gene and belonging to a unique lineage (see blue encircled nodes in Fig. 4) were detected. Analysis of the SPI-1 sequence of the 68 *S*. Derby genomes with truncated *sipA* (Table S2) revealed the presence of an insertion sequence (IS) inside *sipA* belonging to the IS10 subgroup of the IS4 family. Insertion sequences are known to play an important role in bacterial evolution as transposition inside a gene can potentially inactivate it (39). The IS was located at the *sipA* nucleotide position 455/2058, corresponding to amino acid residue 152, upstream of the first fragment along the SipA amino acid sequence (defined as amino acids 294–424) known to have a role in *Salmonella* pathogenesis (14). Consequently, it is very likely that the IS caused the generation of a non-functional protein. In addition, in 49 out of the 68 genomes with truncated *sipA*, a second IS, belonging to the IS10 subgroup of the IS4 family as the one found in *sipA*, was detected inside the *hilD* gene, at nucleotide positions 116/930 and 486/930, corresponding to amino acid positions 39/310 and 162/310, respectively. In both cases, the IS was located upstream of the HilD DNA binding domain, very likely generating a non-functional protein (17). The analysis of SPI-1 in the 11 genomes lacking *sipA* (Table S2) revealed, in 10 genomes, a deletion between nucleotide position 116/930 of *hilD* and nucleotide position 455/2058 of *sipA*. In more detail, the initial part of the same IS previously observed in *sipA* and *hilD* was found downstream of *hilD* nucleotide position 116/930 and its final part was found upstream of *sipA* nucleotide position 455/2058. Notably, the deletion starts and ends at same positions where we observed IS insertions in *sipA* and *hilD* which generated truncated alleles. This observation allows hypothesizing that the deletion was likely the result of the two IS located in *sipA* and *hilD* which mobilized the SPI-1 region located between them. Indeed, IS-related genomic deletions were largely documented in *Salmonella* as well as in many other bacteria (39 – 41). Furthermore, in seven of the genomes carrying an IS4 insertion in *sipA/hilD* genes, a further IS4 insertion was detected in other genes belonging to SPI-1. We next searched for *S*. Derby genomes carrying the observed IS insertion in *sipA* among the 706 ST40 genomes from humans already selected from Enterobase. Only one *S*. Derby genome from humans was found to carry the same insertion (Table S2), strongly indicating that the lineage is rarely represented in *S*. Derby isolated in humans.

Overall these findings, together with those on the stop mutation in *sipA* and *hilC*, demonstrate the existence of *S*. Derby lineages with diverse deleterious mutations in SPI-1 genes, suggesting that distinct evolutionary events resulting in the loss of function of SPI-1 genes occurred in the population of *S*. Derby.

## DISCUSSION

Different serovars of *Salmonella* and different lineages within the same serovar can differ in their host range. An example is represented by the generalist serovar Typhimurium, which includes pathovariants adapted to different hosts (42). Similarly, in a previous study, we identified a specific lineage of *S*. Derby widespread in swine but significantly under-represented in humans compared with the other *S*. Derby lineages, indicating its adaptation to swine associated with reduced virulence to humans. This lineage showed reduced ability to infect enterocytes *in vitro* compared with other *S*. Derby. The reduction was much higher in human cells than in swine cells, and this behavior was due to a loss-of-function mutation in HilD which caused loss of SPI-1 expression. These *in vitro* results, together with the significantly higher presence of that lineage in swine than in humans, suggest that SPI-1 is crucial to cause disease in humans, but not essential for *S*. Derby to circulate in swine, its main host. Accordingly, the literature reports that evolution to host adaptation leads to the loss of functions that are not essential to colonize a specific host (43). Based on this, we further investigated the HilD-impaired lineage detecting two other mutations in SPI-1 genes, namely, a stop mutation in *sipA* and one in *hilC*, indicating that SPI-1 is subjected to genetic decay in this lineage.

The present study shows that the previously characterized lineage, carrying the *hilD* loss-of-function mutation together with *sipA* and *hilC* stop mutations, represents part of a more widespread lineage carrying only the *sipA* and *hilC* stop mutations. This large lineage encompasses c.a. 25% of the *S*. Derby ST40 genomes of swine origin in Enterobase, representing a significant proportion of *S*. Derby from this host. Moreover, this lineage appears to be growing over time among both European and American pigs, while its presence among human cases of salmonellosis is half of that in pigs in both continents. These epidemiological data show that a lineage of *S*. Derby accumulating mutations in genes of SPI-1 is expanding in the swine population and is weakening its virulence to humans. The *in vitro* study of the *hilC*_stop_ and *sipA*_stop_-carrying lineage in human cells shows that introduction of *sipA*_stop_ into a wild-type *S*. Derby reduces cytoplasmic hyper-replication at the level observed for the *hilC*_stop_ and *sipA*_stop_-carrying lineage represented by strain N11. The stop mutation in *sipA* confers the same reduction in cytoplasmic hyper-replication of the deletion of the entire gene, indicating that the missing part of SipA due to the stop mutation is that involved in this phenotype. Cytosolic hyper-replication plays an important role in gut inflammation by inducing extrusion of infected epithelial cells out of the monolayer, inflammatory cell death, and release in the gut lumen of invasion-competent *Salmonella* which infect secondary cells (21). Therefore, the reduced ability of the *hilC*_stop_ and *sipA*_stop_-carrying lineage to hyper-replicate in human cells due to the stop mutation in *sipA* could contribute to the reduced virulence for humans of this lineage.

Regarding the invasion and vacuolar load, N11 has significantly reduced values compared with the wild-type *S*. Derby. N11 showed even lower invasion and vacuolar load than the still-attenuated wild-type strain deleted for both *sipA* and *hilC*, indicating that these phenotypes likely entail also additional genetic features than the observed stop mutations in *sipA* and *hilC*. In line with this, we found non-synonymous/stop mutations other than those in *hilC* and *sipA* in N11 (Table S3). Some mutations were shared by all genomes belonging to the *hilC*_stop_ and *sipA*_stop_-carrying lineage; others were found only in a sub-population of that lineage. Three non-synonymous/stop mutations were located in *citB*, *ssaC*, and *narZ* genes specifically induced in intracellular *S*. Typhimurium (44) and can be involved, likewise *hilC* and *sipA*, in the analyzed phenotypes. The induction of expression of the pro-inflammatory cytokine IL-8 by N11 is significantly reduced compared with wild-type *S*. Derby, and this phenotype could not be fully explained by the tested mutants. Only the *sipA*-deleted mutant, not the mutant carrying *sipA*_stop_, shows reduced IL-8 expression, indicating that the truncated N-terminal domain generated by the stop mutation retains the ability to induce the inflammatory response. The reduced induction of IL-8 expression by N11 requires further investigation.

Overall, these results indicate attenuation for human cells *in vitro* of the lineage of *S*. Derby carrying the stop mutations in *sipA* and *hilC*, which is expanding in the swine host while showing limited virulence to humans.

With regard to swine cells, we observed a progressive decrease in invasion level and vacuolar load moving from *S*. Typhimurium to *S*. Derby without SPI-1 mutations, to *S*. Derby missing *hilC* and *sipA* genes, and to *S*. Derby belonging to the *hilC*_stop_ and *sipA*_stop_-carrying lineage. The latter lineage thus confirms its attenuation also in swine cells. Notably, a strong reduction in invasion and in the vacuolar load was observed in swine cells compared with human cells for all *S*. Derby strains compared with *S*. Typhimurium. In addition, hyper-replication was only measurable for *S*. Typhimurium and not for *S*. Derby strains because of its too low frequency. The infection of the gut mucosa and especially cytosolic hyper-replication are crucial steps in inducing inflammation and thus enteritis; therefore, this *in vitro* evidence is in line with the fact that *in vivo S*. Derby causes asymptomatic infection in swine whereas *S*. Typhimurium can cause enterocolitic forms (36). Our findings in swine cells confirm this previous general knowledge on reduced infectivity of *S*. Derby and add that a widespread lineage belonging to this serovar has even decreased infectivity. This suggests that *S*. Derby does not need to invade the swine intestinal epithelium with high effectiveness in order to circulate in this host and thus can accumulate loss-of-function mutations in genes involved in invasion such as those in SPI-1. As a confirmation of this finding, a second *S*. Derby lineage was found with IS insertion causing truncation and/or loss of one or more SPI-1 genes, indicating that the *S*. Derby population is undergoing converging evolution in the adaptation to swine through loss of function of SPI-1 and the associated attenuation. *Salmonella* can infect and persist in pigs in the intestine, gut-associated lymphoid tissue, and tonsils (37, 45). For example, tonsils are known as an important reservoir of *Salmonella* as it can persist in that compartment for long periods. Boyen et al*.* (45) demonstrated that *S*. Typhimurium SPI-1 genes promote intestinal but not tonsillar colonization in pigs. We therefore hypothesize that the infection of tonsils or other lymphoid districts could be a way for *S*. Derby to persist and spread in pigs.

Overall, this study shows how a host-adapted *Salmonella* serovar, *S*. Derby, is further adapting to its main host, swine, by reducing its virulence in intestinal epithelial cells. The literature reports that *Salmonella* serovars have adapted to specific hosts by losing the ability to replicate in the intestine of those hosts in favor of disseminating to systemic sites (46). However, serovars usually defined as host adapted, like the swine-adapted *S*. Choleraesuis and the bovine-adapted *S*. Dublin, are associated with high virulence, including mortality, in their respective host reservoirs (47) compared with generalist serovars, whereas *S*. Derby causes asymptomatic infection in swine. Therefore, two different routes to host adaptation seem to exist in *Salmonella*, one leading to increased virulence for the target host, the other one leading to attenuation.

## MATERIALS AND METHODS

### Analysis of the structure of the global population of *S*. Derby ST40

The population structure of *S*. Derby ST40 was analyzed by using the whole-genome sequences and tools available in the Enterobase database (https://enterobase.warwick.ac.uk), accessed on 7 April 2023. Specifically, to analyze the whole-genome sequencing data, the allelic profile obtained through core-genome MLST (cgMLST) analysis using the cgMLST V2 + HierCC V1 scheme was used (23). The cgMLST data were obtained in Enterobase by selecting the sequences in the database with the release date preceding the accessed date through the following query: the genomes (i) were assigned to sequence type ST40 using the “Achtman 7 Gene MLST” scheme for *Salmonella*, (ii) had a year of isolation between 2000 and 2023 reported in Enterobase’s “Collection Year” field, (iii) could be assigned to a known country of isolation within the European or North American continents, per Enterobase’s “Country” and “Continent” fields, respectively, and (iv) could be assigned to the source of isolation “Swine” or “Human,” per Enterobase’s “Source Type” and “Source Niche” fields, respectively.

The genomes carrying the described stop mutation in the *sipA* gene were identified through the presence of the allele coded “671” in locus “STMMW_28441” of the cgMLST V2 scheme. The genomes carrying the described stop mutation in the *hilC* gene were identified through the presence of the alleles coded “671” and “835” in locus “STMMW_28291” of the cgMLST V2 scheme. The population tree of *S*. Derby ST40 in swine was constructed in the Enterobase workspace using the GrapeTree option, where the Ninja neighbor-joining algorithm was employed under the cgMLST V2 + HierCC V1 scheme.

We assessed the temporal trend in the occurrence of the investigated mutations in *sipA* and *hilC* genes in the *S*. Derby ST40 population in swine by fitting a generalized linear model with binomial error distribution (logistic regression) using the “Collection Year” and the “Continent” as explanatory variables. Moreover, we assessed whether the occurrence of the investigated mutations in *sipA* and *hilC* genes is different between *S*. Derby isolated in swine and *S*. Derby isolated in human by fitting a logistic regression using the source of isolation and the continent as explanatory variables.

The detection of other lineages of *S*. Derby carrying potentially deleterious mutations in *hilC* and *sipA* gene was performed investigating the allelic variants observed in the “STMMW_28291” corresponding to *hilC* and “STMMW_28441” corresponding to *sipA* loci of the cgMLST V2 scheme within the population of the ST40 genomes extracted from Enterobase. Since we were interested in detecting potentially deleterious mutations in the *sipA* gene that were reasonably diffused in the ST40 population, we genetically characterized only the allelic variants in locus “STMMW_28441” observed in more than the 5% of the genomes. We considered as deleterious mutations missense mutations, stop mutations, and mutations generating a truncated or missing allele.

### Bacterial strains

Bacterial strains used in this study are listed in Table S4. Bacteria were cultured in Luria Bertani (LB) Miller medium supplemented with appropriate antibiotics (ampicillin 100 µg/mL, kanamycin 50 µg/mL, and chloramphenicol 20 µg/mL) when needed.

### Construction of recombinant strains

Plasmids and primers used in this study are listed in Tables S5 and S6, respectively. Gene deletion and allelic exchange were made using the bacteriophage λ red recombinase system (48). For gene deletion, genes were replaced with a kanamycin or chloramphenicol resistance cassette (*kan* or *cat*) amplified from pKD4 and pKD3 template plasmids, respectively. For allelic exchange, constructs were generated by overlap PCR to contain *sipA*_stop_ and the *kan* resistance cassette from the pKD4 plasmid. Overlap PCR and transformation were made according to Tambassi et al. (5).

### Mammalian cell cultures

INT-407 embryonic human intestine, HeLa derivative, epithelial cell line, and IPEC-J2 non-transformed swine intestinal epithelial cell line were purchased from the OIE collaborating Italian Biobank of Veterinary Resources. INT-407 cells were cultured in Minimum Essential Medium (MEM; Sigma-Aldrich) containing 10% fetal bovine serum, penicillin 100 U/mL, and streptomycin 100 µg/mL (pen/strep). IPEC-J2 cells were cultured in 50% Dulbecco’s modified Eagle’s medium (DMEM, high glucose; Sigma-Aldrich) and 50% Ham’s F12 Nutrient Mixture (Sigma-Aldrich) containing 5% fetal bovine serum, supplemented with pen/strep. Both cell lines were maintained at 37°C in 5% CO_2_ and used within 12 passages of receipt.

### Automated analysis of *Salmonella* intracellular phenotypes

The bacterial infection of INT-407 and IPEC-J2 cells for the quantification of *Salmonella* intracellular phenotypes was performed according to Berni et al. (25). Briefly, INT-407 and IPECJ-2 cells were seeded at a density of 3 × 10^4^ and 1 × 10^4^ cells/well, respectively, in antibiotic-free media 20–24 hours prior to infection in 96-well imaging plates (CellVis) coated with collagen I from rat tail (Invitrogen). *Salmonella* strains used for the infection were transformed with the pCHAR-Duo reporter plasmid that was kindly provided by Dr. Olivia Steele-Mortimer. The pCHAR-Duo plasmid allows to distinguish vacuolar from cytosolic *Salmonella* through the differential expression of mCherry and GFP (49). *Salmonella* strains were cultured statically for 20 hours at 37°C in LB supplemented with ampicillin 100 µg/mL to reach the stationary phase of growth. Then, 100% of confluent monolayers were washed with PBS and infected for 1 hour with ~1.5 × 10^5^ bacteria/mm^2^ at 37°C in 5% CO_2_ with a breathable sealing membrane (Diversified Biotech BEM-1). A multiplicity of infection (MOI) based on the growth surface, rather than on the epithelial cell number, was applied to expose INT-407 and IPEC-J2 confluent monolayers to the same bacterial load, despite the different size of the two cell lines. After 1 hour of infection, monolayers were washed with PBS, treated with gentamicin 100 µg/mL for 1 hour, then washed again with PBS, and treated with gentamicin 10 µg/mL for the remaining time course of the infection.

After 8 hours of infection, monolayers were washed with PBS and fixed with paraformaldehyde 4% (Sigma-Aldrich) for 20 min at room temperature. Finally, epithelial cells were labelled with the HCS CellMask Blue cytoplasmic⁄nuclear stain (Invitrogen) following the manufacturer’s instructions.

Samples were imaged with a motorized Axio Observer Inverted Microscope with Colibri 5/7 light source (ZEISS) using 40×/0.75 NA objective for INT-407 and 20×/0.8 NA objective for IPEC-J2 because the two cell lines have different size. At least 1,000 epithelial cells were pictured for each biological replicate. The automated fluorescence image analysis was performed with FIJI (NIH) (50). The image analysis workflow and scripts for the automation were described in detail by Berni et al. (25). Briefly, epithelial cells were first segmented and defined as a region of interest (ROI), uniquely labelled with their *y*-*x* coordinates. Then, the area and the percentage of the ROI area occupied by *Salmonella* expressing the mCherry constitutive reporter only or also the GFP cytosol-responsive reporter were measured for each ROI. The percentage of the cell area occupied by mCherry-only expressing *Salmonella* was used to calculate the percentage of infected cells (considering only ROIs with a percentage of occupied area > 0.2%) and the mean vacuolar load. The percentage of the cell area occupied by GFP-expressing *Salmonella* was measured to quantify the cytosolic hyper-replication rate scored as the fraction of infected cells massively colonized by cytosolic *Salmonella* (considering only cells with a percentage of the occupied area ≥20% and ≥15% for human and swine cells, respectively). The statistical analysis on the microscopy data (i.e., fraction of infected cells, vacuolar load, and hyper-replication rate) was performed by using linear mixed models where the samples act as fixed variables and the technical repetitions within the same biological repetition act as random effects (pseudo-replication). The *P*-values were obtained from the *t*-statistics using Satterthwaite’s method with Bonferroni post-hoc correction.

### RNA extraction and cDNA synthesis

RNA was extracted from *Salmonella* strains grown in *in vitro* SPI-1-inducing conditions and from epithelial cells infected by *Salmonella* strains.

To reach the SPI-1-inducing conditions *in vitro*, *Salmonella* strains were grown in 2-mL LB broth for 18 hours at 37°C at 220 rpm, then diluted 300 µL in 10 mL of fresh LB, and grown at 37°C at 200 rpm for ~2 hours until the early stationary phase of growth was reached (corresponding to OD_600_ = 2.0) (5, 24). Then, 500 µL of bacteria culture was treated with RNA protect (Qiagen) and RNA was extracted using the Nucleospin RNA extraction kit (Macherey Nagel) following the manufacturer’s instructions.

For RNA extraction from *Salmonella*-infected cells, INT-407 and IPEC-J2 were cultured in antibiotic-free media 20–24 hours prior to infection in 24-well plates coated with collagen I from rat tail (Invitrogen). Seeding densities of 3 × 10^5^ and 1.5 × 10^5^ cells/well were used to reach 100% confluency. *Salmonella* isolates were grown statically for 20 hours at 37°C in LB broth. Confluent monolayers were washed with PBS and infected with a MOI based on the growth surface as described previously. After 1 hour of infection, the inoculum was removed and fresh medium was added until the end of the infection (33). After 4 hours of infection, medium was removed and RNA was extracted using the Nucleospin RNA extraction kit (Macherey Nagel) by immediately adding 350 μL of RA1, following the manufacturer’s protocol.

For both bacterial and eukaryotic-extracted RNA, in-column and in-solution DNase digestion was performed following Nucleospin RNA extraction kit protocol. Then, RNA was precipitated by mixing with sodium acetate (0.1 vol, 3M), ethanol (2.5 vol, 100%), and glycogen (5 µg/sample). After overnight precipitation at −20°C, RNA was washed twice with 750 µL of 70% ice-cold ethanol and resuspended in nuclease-free water. RNA concentration and quality (A_260_/A_280_) were determined with a Synergy H1 Hybrid spectrophotometer (BioTek) equipped with Gen5 software. The extracted RNA was reverse transcribed using the High-Capacity cDNA Reverse Transcription Kit (Applied Biosystems).

### Gene expression analysis

Gene expression analyses were performed by quantitative real-time PCR (qRT-PCR) using the kit GoTaq qPCR Master Mix (Promega Corporation) and the Biorad CFX-96 thermocycler. Each reaction was performed in two technical replicates for each sample. Primer sequences are indicated in Table S5.

The differential expression of eight SPI-1 genes representative for transcriptional regulators, effectors, needle-complex structural component, and chaperone was calculated for ER1175ΔhilC and N11 vs ER1175. Expression of target genes was normalized to the reference gene *gmk*.

INT-407 cells infected by *Salmonella* strains were analyzed for IL-8 expression. Expression of IL-8 was normalized to the reference gene *GADPH*. Uninfected epithelial cells were used as control condition. The “Common Base Method” described by Ganger et al. (51) was used for the analysis of qRT-PCR data. Briefly, the Ct values were normalized for the reaction efficiency (E) as follows: *log2(E)·Ct*. Differences in gene expression among samples were analyzed using unpaired *t*-test on efficiency-weighted ΔCt assuming unequal variances.

### Bioinformatic analysis

*S*. Derby genome assemblies with missing or truncated *sipA* according to the allele calling of cgMLST were downloaded from Enterobase. Contigs of each *S*. Derby were aligned to the SPI-1 sequence of *S*. Typhimurium SL1344 (GenBank FQ312003.1, from *sitA* CDS to *invH* CDS) using MAUVE through Geneious 11.1.5 (https://www.geneious.com) to visualize the *sipA* gene in the context of the whole SPI-1 sequence. The nucleotide sequences found inserted in the *sipA* gene and in other SPI-1 genes were subjected to BLASTN using the ISfinder database (http://www-is.biotoul.fr) (52).

## Data Availability

All supporting data and protocols have been provided within the article or through supplementary data files. Data from Enterobase are publicly available.

## SUPPLEMENTAL MATERIAL

Supplemental Material spectrum.01899-23-s0001.docx Fig S1 and Fig S2, Tables S1 to S6.

ASM does not own the copyrights to Supplemental Material that may be linked to, or accessed through, an article. The authors have granted ASM a non-exclusive, world-wide license to publish the Supplemental Material files. Please contact the corresponding author directly for reuse.
