# Supplementary material for: Salmonella Derby adaptation to swine and simultaneous attenuation for humans go through decay of Salmonella Pathogenicity Island I
Source: Microbiol Spectr. 2023 Oct 6;11(6):e01899-23. doi: 10.1128/spectrum.01899-23 (PMC10715017; doi:10.1128/spectrum.01899-23)
Supplement: Supplemental Material — Fig S1 and Fig S2, Tables S1 to S6. [file spectrum.01899-23-s0001.docx]

# ***Salmonella* Derby adaptation to swine and simultaneous attenuation for humans goes through decay of *Salmonella* Pathogenicity Island I**

**Running title: Decay of *Salmonella* Pathogenicity Island I in *Salmonella* Derby**

Melissa Berni^a^, Luca Bolzoni^a^, Ilaria Menozzi^a^, Alessandra Dodi^a^, Chiara Bracchi^a^, Marina Morganti^a^, Erika Scaltriti^a^, Stefano Pongolini^a^, Martina Tambassi^a#^

**Affiliation**

^a^ Risk Analysis and Genomic Epidemiology Unit, Istituto Zooprofilattico Sperimentale della Lombardia e dell'Emilia-Romagna (IZSLER), Parma, Italy

# Address correspondence to Martina Tambassi, martina.tambassi@izsler.it

Melissa Berni and Luca Bolzoni contributed equally to this work.

**Supplementary material**

This file contains:

Fig S1

Fig S2

Table S1

Table S2

Table S3

Table S4

Table S5

Table S6


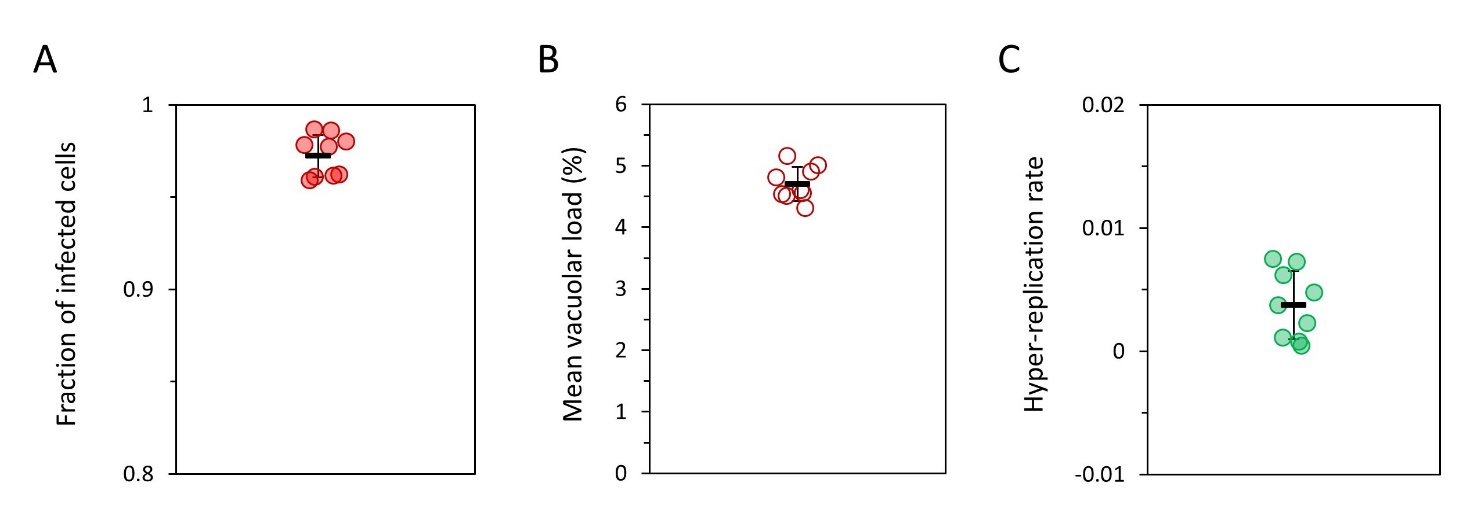


**FIG S1** *S*. Typhimurium SL1344 single-cell quantification of the fraction of infected cells (A), the mean vacuolar load (B) and the hyper-replication rate (C) in human cells. Data of three biological replicates with three technical replicates each are reported. Horizontal bars indicate the mean between biological replicates. Vertical bars indicate standard deviation. At least 3000 human cells were analyzed per biological replicate.

**
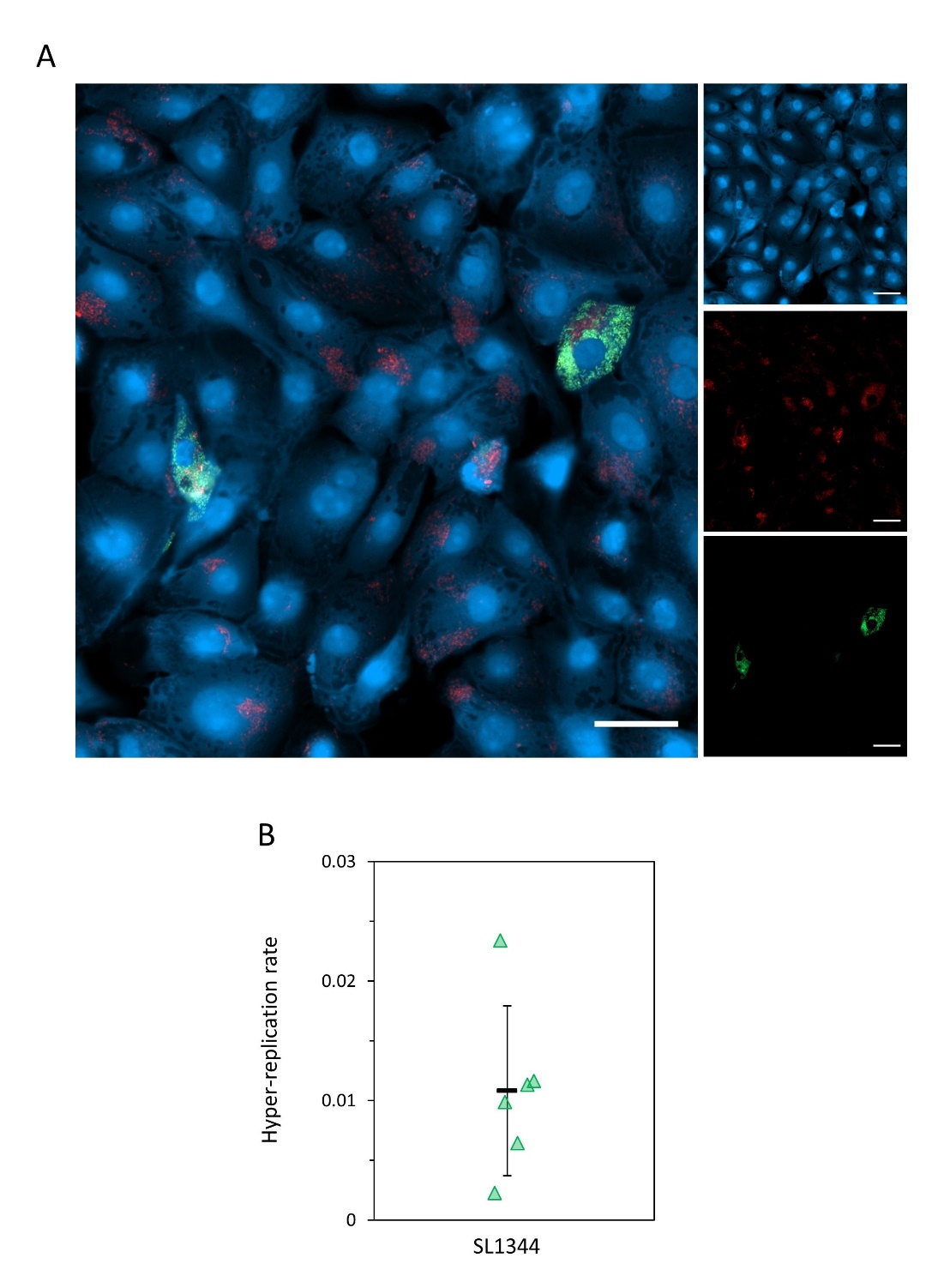
**

**FIG S2** *S*. Typhimurium SL1344 single-cell quantification of hyper-replication rate in swine cells. (A) Representative image of swine epithelial cells infected with SL1344 carrying the pCHAR-Duo fluorescence reporter plasmid. Epithelial cells are shown in blue (HCS CellMask Blue), intracellular Salmonellae in red (mCherry), cytosolic hyper-replicating Salmonellae in green (GFP). 20x/0.8 NA objective was used. Image is a Region of Interest (ROI) from a 4x4 tile region. White scale bars are 50 µm. (B) The hyper-replication rate was calculated by dividing the number of cells with a percentage of area occupied by GFP-expressing Salmonellae ≥15% by the total number of infected cells. Each dot represents a technical replicate. Horizontal bar indicates the mean between biological replicates. Vertical bar indicates standard deviation. Data from two biological replicates with three technical replicates each are reported.

**Table S1** SPI-1 genes expression under *in vitro* inducing conditions. Quantitative RT-PCR was performed on eight SPI-1 genes on ER1175, ER1175Δ*hilC* and N11 strains. The mean fold change of three biological replicates of N11 and ER1175ΔhilC versus ER1175 are reported.


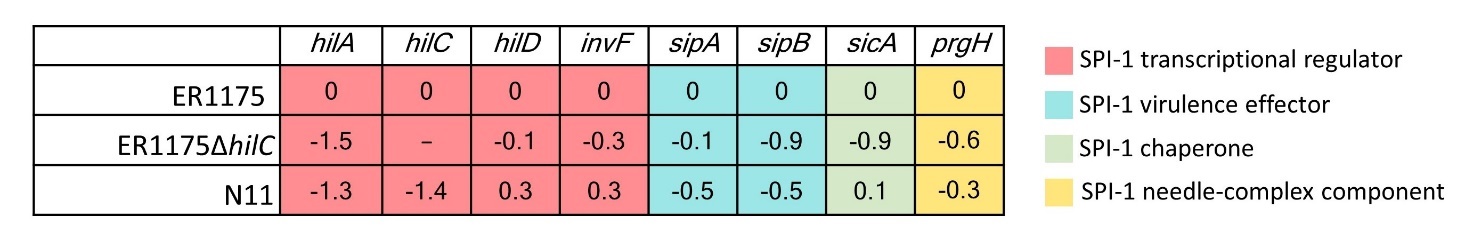


**Table S2** List of *S*. Derby genomes with *sipA* truncated or missing according to the allele-calling of core genome MLST performed by Enterobase**.** For each *S*. Derby genome, the sample name, the source of isolation, the collection year, the isolation country, the *sipA* allele according to the cgMLST V2 scheme (STMMW_28441 sipA: “-1” indicates truncated allele; “-“ indicates missing allele), the position of IS4 insertion in *sipA* and *hilD* and other modifications on SPI-1 sequence were indicated.

| Name | Source | Collection Year | IsolationCountry | *sipA* allele | position of IS4 in *sipA* | position of IS4 in *hilD* | Other mutations in SPI-1 |
| --- | --- | --- | --- | --- | --- | --- | --- |
| FSIS12320564 | Swine | 2023 | United States | -1 | 455/2058 | 116/930 |  |
| FSIS32104879 | Swine | 2021 | United States | -1 | 455/2058 | 116/930 |  |
| FSIS11919874 | Swine | 2019 | United States | -1 | 455/2058 | 116/930 |  |
| FSIS11920773 | Swine | 2019 | United States | -1 | 455/2058 | 116/930 |  |
| FSIS11921153 | Swine | 2019 | United States | -1 | 455/2058 | 116/930 |  |
| FSIS31902534 | Swine | 2019 | United States | -1 | 455/2058 | 116/930 |  |
| FSIS11923793 | Swine | 2019 | United States | -1 | 455/2058 | 116/930 |  |
| FSIS11925911 | Swine | 2019 | United States | -1 | 455/2058 | 116/930 | IS4 insertion in *sipB* |
| FSIS11926575 | Swine | 2019 | United States | - | 455/2058 | 116/930 | SPI-1 region between *hilD* and *sipA* missing; insertion in *prgK* |
| FSIS11816500 | Swine | 2018 | United States | -1 | 455/2058 | 116/930 |  |
| FSIS21823025 | Swine | 2018 | United States | -1 | 455/2058 | 116/930 |  |
| FSIS1710224 | Swine | 2017 | United States | -1 | 455/2058 | 116/930 |  |
| FSIS1710346 | Swine | 2017 | United States | -1 | 455/2058 | 116/930 |  |
| FSIS1710861 | Swine | 2017 | United States | -1 | 455/2058 | 116/930 |  |
| FSIS1710870 | Swine | 2017 | United States | -1 | 455/2058 | 116/930 |  |
| FSIS1710212 | Swine | 2017 | United States | -1 | 455/2058 | 116/930 |  |
| FSIS1700245 | Swine | 2017 | United States | -1 | 455/2058 | 116/930 |  |
| FSIS1700948 | Swine | 2017 | United States | -1 | 455/2058 | 116/930 | IS4 insertion in *prgH* |
| CVM N17S429 | Swine | 2017 | United States | -1 | 455/2058 | 116/930 |  |
| FSIS1606325 | Swine | 2016 | United States | -1 | 455/2058 | 116/930 |  |
| FSIS1709855 | Swine | 2016 | United States | - | 455/2058 | 116/930 | SPI-1 region between *hilD* and *sipA* missing |
| CVM N58036 | Swine | 2015 | United States | -1 | 455/2058 | 116/930 |  |
| FSIS1502420 | Swine | 2015 | United States | -1 | 455/2058 | 116/930 |  |
| FSIS32104937 | Swine | 2021 | United States | -1 | 455/2058 | no insertion |  |
| FSIS12032100 | Swine | 2020 | United States | - | 455/2058 | 116/930 | SPI-1 region between *hilD* and *sipA* missing |
| FSIS11807373 | Swine | 2018 | United States | -1 | 455/2058 | no insertion |  |
| FSIS11811970 | Swine | 2018 | United States | -1 | 455/2058 | 116/930* |  |
| SAL-22-VL-ON-MB-0004 | Swine | 2022 | Canada | -1 | 455/2058 | 116/930 |  |
| CVM N20S0277 | Swine | 2020 | United States | -1 | 455/2058 | 116/930 |  |
| FSIS22206973 | Swine | 2022 | United States | -1 | 455/2058 | 116/930 |  |
| FSIS22130496 | Swine | 2021 | United States | - | 455/2058 | 116/930 | SPI-1 region between *hilD* and *sipA* missing |
| FSIS32105112 | Swine | 2021 | United States | -1 | 455/2058 | 116/930 | IS4 insertion in *sipB* |
| FSIS12028857 | Swine | 2020 | United States | - | 455/2058 | 116/930 | SPI-1 region between *hilD* and *sipA* missing |
| FSIS12030710 | Swine | 2020 | United States | -1 | 455/2058 | 116/930 |  |
| FSIS12031546 | Swine | 2020 | United States | - | 455/2058 | 116/930 | SPI-1 region between *hilD* and *sipA* missing |
| FSIS32003724 | Swine | 2020 | United States | -1 | 455/2058 | 116/930 |  |
| FSIS11924005 | Swine | 2019 | United States | -1 | 455/2058 | 116/930 |  |
| FSIS11812713 | Swine | 2018 | United States | -1 | 455/2058 | 116/930 |  |
| FSIS11815719 | Swine | 2018 | United States | -1 | 455/2058 | 116/930 |  |
| FSIS11816981 | Swine | 2018 | United States | -1 | 455/2058 | 116/930 |  |
| FSIS1710981 | Swine | 2017 | United States | -1 | 455/2058 | 116/930 |  |
| FSIS1701590 | Swine | 2017 | United States | -1 | 455/2058 | 116/930 |  |
| FSIS1703656 | Swine | 2017 | United States | -1 | 455/2058 | 116/930 |  |
| FSIS1607970 | Swine | 2016 | United States | - | 455/2058 | 116/930 | SPI-1 region between hilD and sipA missing |
| CVM N56916F | Swine | 2014 | United States | -1 | 455/2058 | 116/930 |  |
| CVM N57792F | Swine | 2014 | United States | -1 | 455/2058 | 116/930 |  |
| CVM N57811F | Swine | 2014 | United States | - | 455/2058 | 116/930 | SPI-1 region between *hilD* and *sipA* missing |
| CVM N57961F | Swine | 2014 | United States | - | 455/2058 | 116/930 | SPI-1 region between *hilD* and *sipA* missing |
| CVM N54262 | Swine | 2014 | United States | -1 | 455/2058 | 116/930 |  |
| FSIS12210504 | Swine | 2022 | United States | -1 | 455/2058 | no insertion |  |
| FSIS12217633 | Swine | 2022 | United States | -1 | 455/2058 | no insertion | IS4 insertion in *prgH* |
| FSIS22130571 | Swine | 2021 | United States | -1 | 455/2058 | no insertion |  |
| FSIS12139612 | Swine | 2021 | United States | -1 | 455/2058 | no insertion |  |
| FSIS22029485 | Swine | 2020 | United States | -1 | 455/2058 | no insertion |  |
| FSIS21923310 | Swine | 2019 | United States | -1 | 455/2058 | no insertion |  |
| FSIS11926992 | Swine | 2019 | United States | -1 | 455/2058 | no insertion |  |
| FSIS21720146 | Swine | 2017 | United States | -1 | 455/2058 | no insertion |  |
| FSIS1703405 | Swine | 2017 | United States | -1 | 455/2058 | no insertion |  |
| FSIS1606689 | Swine | 2016 | United States | -1 | 455/2058 | no insertion |  |
| FSIS1608420 | Swine | 2016 | United States | -1 | 455/2058 | no insertion |  |
| ADRDL-261 | Swine | 2016 | United States | -1 | 455/2058 | 116/930 |  |
| FSIS22312380 | Swine | 2023 | United States | -1 | 455/2058 | 486/930 |  |
| FSIS32208000 | Swine | 2022 | United States | - | 455/2058 | 116/930 | SPI-1 region between *hilD* and *sipA* missing |
| FSIS32105848 | Swine | 2021 | United States | -1 | 455/2058 | 486/930 |  |
| FSIS12027978 | Swine | 2020 | United States | -1 | 455/2058 | 116/930 |  |
| FSIS1710169 | Swine | 2017 | United States | -1 | 455/2058 | 116/930 |  |
| FSIS1700527 | Swine | 2017 | United States | -1 | 455/2058 | 116/930 |  |
| FSIS1702822 | Swine | 2017 | United States | -1 | 455/2058 | 116/930 |  |
| FSIS1702854 | Swine | 2017 | United States | -1 | 455/2058 | 116/930 |  |
| FSIS1608829 | Swine | 2016 | United States | -1 | 455/2058 | 116/930 |  |
| FSIS1609345 | Swine | 2016 | United States | -1 | 455/2058 | 116/930 |  |
| FSIS1703081 | Swine | 2017 | United States | -1 | 455/2058 | no insertion | IS4 insertion downstream *hilC* |
| FSIS32104541 | Swine | 2020 | United States | -1 | 455/2058 | no insertion |  |
| FSIS11812167 | Swine | 2018 | United States | -1 | 455/2058 | no insertion |  |
| FSIS22131484 | Swine | 2021 | United States | -1 | 455/2058 | no insertion |  |
| FSIS12031944 | Swine | 2020 | United States | -1 | 455/2058 | no insertion |  |
| FSIS12140649 | Swine | 2021 | United States | -1 | 455/2058 | no insertion | IS4 insertion in *orgC* |
| FSIS22207943 | Swine | 2022 | United States | - | *sipA* missing | *hilD* missing | SPI-1 region between *prgH* and *invH* missing |
| FSIS12320029 | Swine | 2023 | United States | -1 | 455/2058 | 116/930 |  |
| S14_0301 | Swine | 2014 | Poland | -1 | 368/2058 | no insertion | IS4 insertion downstream *hilD* and *hilC* |
| NL_L3 | Swine | 2006 | Ireland | -1 | 368/2058 | no insertion | IS4 insertion downstream *hilD* |
| NL_L4 | Swine | 2006 | Ireland | -1 | 368/2058 | no insertion | IS4 insertion downstream *hilD* |
| NL_L7 | Swine | 2006 | Ireland | -1 | 368/2058 | no insertion | IS4 insertion downstream *hilD* |
| ADRDL-2889 | Swine | 2019 | United States | -1 | 605/2058 | no insertion |  |
| PNUSAS004667 | Human | 2016 | United States | -1 | 455/2058 | 116/930 |  |

**Table S3** List of N11 nonsynonymous and stop mutations. For each mutation, the gene, the product, the *S*. Typhimurium locus tag of the gene, the position inside the gene, its effect on the nucleotide and amino acid sequence and its presence in all genomes of the S. Derby *hilC_stop_ and sipA_stop_* -carrying lineage (indicated by a “✓”) were reported. Mutations were detected by analysing the core Single Nucleotide Polymorphisms (SNPs) alignment generated in Tambassi et *al*, 2020 (1). Mutations shared by N11 and genomes belonging to the *hilD*-mutated lineage (part of the *hilC_stop_ and sipA_stop_* -carrying lineage) but not by ER1175 and the other analysed genomes were reported.

| **Gene** | **Product** | **SL1344**  **locus tag** | **SNP Position in CDS (5' -> 3')** | **Effect** | **Presence in all *hilC_stop_ and sipA_stop_* lineage** |
| --- | --- | --- | --- | --- | --- |
| *yadB* | glutamyl-tRNA synthetase-related protei | SL1344_0186 | 418/942 | nonsynonymous  c.418G>A p.Gly140Arg |  |
| *sciB* | conserved hypothetical protein (SPI-6 associated) | SL1344_0261 | 728/996 | nonsynonymous  c.728G>A p.Arg243Gln |  |
| *rhs* | Rhs-family protein | / | 257/2463 | nonsynonymous  c.257A>G p.Asp86Gly | ✓ |
| *citG* | triphosphoribosyl-dephospho-CoA synthase | SL1344_0607 | 881/894 | nonsynonymous  c.881A>G p.Gln294Arg |  |
| *citB* | transcriptional regulatory protein | SL1344_0614 | 1636/1662 | nonsynonymous c.1636A>G p.Arg546Gly | ✓ |
| *fliC* | flagellin | SL1344_1888 | 736/1518 | nonsynonymous  c.736G>A p.Asp246Asn |  |
| *yejH* | hypothetical helicase | SL1344_2200 | 470/1761 | nonsynonymous  c.470G>A p.Gly157Glu |  |
| *yehU* | hypothetical two-component system sensor kinase | SL1344_2137 | 1495/1686 | nonsynonymous  c.1495C>T p.His499Tyr |  |
| *yceO* | conserved hypothetical protein | SL1344_1096 | 23/114 | nonsynonymous  c.23T>A p.Leu8Gln |  |
| *fabF* | 3-oxoacyl-[acyl-carrier-protein] synthase II | SL1344_1134 | 962/1242 | nonsynonymous  c.962C>T p.Ser321Leu |  |
| *ssaC* | outer membrane secretory protein | SL1344_1328 | 1153/1494 | nonsynonymous c.1153G>A p.Val385Ile |  |
| *narZ* | respiratory nitrate reductase 2 alpha chain | SL1344_1508 | 2215/2217 | stop  c.2215C>T p.Gln739* | ✓ |
| *gatC* | hypothetical phosphotransferase enzyme | SL1344_1544 | 40/1314 | nonsynonymous  c.40G>A p.Val14Met | ✓ |
| *yciK* | hypothetical oxidoreductase | SL1344_1649 | 493/762 | nonsynonymous  c.493T>A p.Tyr165Asn |  |
| *arnT* | melittin resistance protein PqaB | SL1344_2270 | 610/1647 | nonsynonymous  c.610G>A p.Asp204Asn |  |
| *hycE* | formate hydrogenlyase subunit 5 | SL1344_2829 | 1529/1710 | nonsynonymous c.1529G>A p.Arg510His |  |
| *hilC* | AraC-family transcriptional regulator | SL1344_2847 | 626/627 | stop  c.626G>A p.Trp209* | ✓ |
| *sipA* | pathogenicity island 1 Type III secretion system effector protein | SL1344_2861 | 1006/1008 | stop  c.1006G>T p.Glu336* | ✓ |
| *rtcR* | transcriptional regulatory protein. | SL1344_3488 | 133/1584 | nonsynonymous  c.133G>A p.Glu45Lys |  |
| *gtrCb* | hypothetical membrane protein | SL1344_4139 | 737/1539 | nonsynonymous  c.737C>A p.Thr246Lys | ✓ |
| *yjiO* | hypothetical sugar transport protein | SL1344_4448 | 625/1242 | nonsynonymous  c.625T>C p.Arg209Cys |  |

**Table S4** Strains used in the study.

| **Name** | **Serovar** | **Genotype** | **Reference** |
| --- | --- | --- | --- |
| ER1175 | Derby | Wildtype | [1] |
| ER1175::s*ipA_stop_* | Derby | Δ*sipA*::*sipA_stop_-kan* | This study |
| ER1175Δ*sipA* | Derby | Δ*sipA*::*kan* | This study |
| ER1175Δ*hilC* | Derby | Δ*hilC*::*kan* | This study |
| ER1175Δ*sipA*Δ*hilC* | Derby | Δ*sipA::kan*, Δ*hilC::cat* | This study |
| N11 | Derby | Wildtype | [1] |
| SL1344 | Typhimurium | Wildtype | National Collection of Type Cultures (NCTC) |

**Table S5** Plasmids used in the study.

| **Name** | **Reference** |
| --- | --- |
| pKD46 | [2] |
| pKD4 | [2] |
| pKD3 | [2] |
| pCHAR1 | [3] |

**Table S6** Primers used in the study.

| **Primer Name** | **Sequence 5' -> 3'** | **Purpose** | **Reference** |
| --- | --- | --- | --- |
| kan_KO_C_FW | CGGTGCCCTGAATGAACTGC | Control for *kan-*cassette insertion, *kan*-specific primer | [2] |
| sipA_KO_FW | AACAGAAGAGGATATTAATAATGGTTACAAGTGTAAGGTGTAGGCTGGAGCTGCTTCG | *sipA* knockout, *kan* insertion | [4] |
| sipA_KO_RV | CTTTCCCGGTTAATTAACGCTGCATGTGCAAGCCATCAACGCATATGAATATCCTCCTTAG | *sipA* knockout, *kan* insertion | [4] |
| sipA_KO_C_RV | CGCAAAGGTCGTCATATCAGG | Control for *kan* cassette insertion, *sipA*-specific primer | This study |
| hilC_KO_FW | TATAACGATTTTGAGTTCCTTATAGCACACAGGATAAAATTGTAGGCTGGAGCTGCTTCG | *hilC* knockout, *kan* insertion | This study |
| hilC_KO_RV | TAACGCAGACAGATAGTAACGTTTAAAATAATTTCACAAACATATGAATATCCTCCTTAG | *hilC* knockout, *kan* insertion | This study |
| hilC_KO_C_RV | TTCTTCCTGGGCGACTACTG | Control for *kan* and cat cassette insertion, *hilC*-specific primer | This study |
| cat_KO_C_FW | CCGTTTTCACCATGGGCAAA | Control for *cat* cassette insertion, *cat*C-specific primer | This study |
| hilC_KO_cat_FW | TATAACGATTTTGAGTTCCTTATAGCACACAGGATAAAATGCCTACCTGTGACGGAAGAT | *hilC* Knockout, *cat* insertion | This study |
| hilC_KO_cat_RV | TAACGCAGACAGATAGTAACGTTTAAAATAATTTCACAAAGTGTAGGCTGGAGCTGCTTC | *hilC* Knockout, *cat* insertion | This study |
| sipA_SNP_1_FW | GGGAAATTAAAGGCCCGCAA | *sipA*-*kan* Overlap-PCR | This study |
| sipA_SNP_2_RV | CCAGCCTACATTAACGCTGCATGTGCAAGC | *sipA*-*kan* Overlap-PCR | This study |
| sipA_SNP_3_FW | CAGCGTTAATGTAGGCTGGAGCTGCTTCG | *sipA-kan* Overlap-PCR | This study |
| sipA_SNP_4_RV | TTGCTTCAATATCCATATTCATCGCATCTTTCCCGGTTAACATATGAATATCCTCCTTAG | *sipA-kan* Overlap-PCR | This study |
| sipA_SNP_FW | ACAGCCAACGCCATGGTG | *sipA* specific PCR | This study |
| sipAstop_SNP_FW | ACAGCCAACGCCATGGTT | *sipA_stop_* specific PCR | This study |
| gmk_RTPCR_FW | TTGGCAGGGAGGCGTTT | Gene expression analysis | [1] |
| gmk_RTPCR_RV | GCGCGAAGTGCCGTAGTAAT | Gene expression analysis | [1] |
| hilD_RTPCR_FW | GGCGCTCTCTATGCACTTATC | Gene expression analysis | [1] |
| hilD_RTPCR_RV | GCAGGAAAGTCAGGCGTATAG | Gene expression analysis | [1] |
| hilA_RTPCR_FW | ATCGTCGGGAGTTTGCTATTC | Gene expression analysis | [1] |
| hilA_RTPCR_RV | CTGACCAGCCATGAAGAGATT | Gene expression analysis | [1] |
| hilC_RTPCR_FW | TGTCCACGGGTTTGTAGTAATG | Gene expression analysis | [1] |
| hilC_RTPCR_RV | TGCTCGCTCAAGGAAATCAA | Gene expression analysis | [1] |
| rtsA_RTPCR_FW | CAGGTGGGGAGCATTGAATG | Gene expression analysis | [1] |
| rtsA_RTPCR_RV | GGTGAGCTTGATGAGTACGG | Gene expression analysis | [1] |
| invA_RTPCR_FW | TGGAGCATATTCGTGGAGCA | Gene expression analysis | [1] |
| invA_RTPCR_RV | AGGTCTGACGGATCCCTTTG | Gene expression analysis | [1] |
| invF_RTPCR_FW | AGAAGGCCACGAGAACATCA | Gene expression analysis | [1] |
| invF_RTPCR_RV | GAAACGCCGATCAGCTCTTT | Gene expression analysis | [1] |
| sipB_RTPCR_FW | GCAAAATGATGGGCGAAACG | Gene expression analysis | [1] |
| sipB_RTPCR_RV | CATTACCCAGGCCGCTAGTA | Gene expression analysis | [1] |
| IL8-FW | CATACTCCAAACCTTTCCACCCC | Gene expression analysis | [5] |
| IL8-RV | TCAGCCCTCTTCAAAAACTTCTCCA | Gene expression analysis | [5] |
| GADPH-FW | TCAAGGCTGAGAACGGGAAG | Gene expression analysis | [6] |
| GADPH-RV | CGCCCCACTTGATTTTGGAG | Gene expression analysis | [6] |

## Supplementary references

1. Tambassi M, Berni M, Bracchi C, Scaltriti E, Morganti M, Bolzoni L, Tanner JR, Thilliez G, Kingsley RA, Pongolini S, Casadei G. 2020. Mutation of *hilD* in a *Salmonella* Derby lineage linked to swine adaptation and reduced risk to human health. Sci Rep 10(1):21539. <https://doi.org/10.1038/s41598-020-78443-7>

2. Datsenko KA, Wanner BL. 2000. One-step inactivation of chromosomal genes in Escherichia coli K-12 using PCR products. Proc Natl Acad Sci U S A 97(12):6640-5. <https://doi.org/10.1073/pnas.120163297>

3. Cooper KG, Chong A, Starr T, Finn CE, Steele-Mortimer O. 2017. Predictable, Tunable Protein Production in *Salmonella* for Studying Host-Pathogen Interactions. Front Cell Infect Microbiol 7:475. <https://doi.org/10.3389/fcimb.2017.00475>

4. Finn CE, Chong A, Cooper KG, Starr T, Steele-Mortimer O. 2017. A second wave of Salmonella T3SS1 activity prolongs the lifespan of infected epithelial cells. PLoS Pathog 13(4):e1006354. <https://doi.org/10.1371/journal.ppat>

5. Xu L, Feng X, Tan W, Gu W, Guo D, Zhang M, Wang F. 2013, IL-29 enhances Toll-like receptor-mediated IL-6 and IL-8 production by the synovial fibroblasts from rheumatoid arthritis patients. Arthritis Res Ther 15(5):R170. <https://doi.org/10.1186/ar4357>

6. Dahn ML, Dean CA, Jo DB, Coyle KM, Marcato P. 2020. Human-specific GAPDH qRT-PCR is an accurate and sensitive method of xenograft metastasis quantification. Mol Ther Methods Clin Dev 20:398-408.  <https://doi.org/10.1016/j.omtm.2020.12.010>
